# Potential Hazard of Lanthanides and Lanthanide-Based Nanoparticles to Aquatic Ecosystems: Data Gaps, Challenges and Future Research Needs Derived from Bibliometric Analysis

**DOI:** 10.3390/nano10020328

**Published:** 2020-02-14

**Authors:** Irina Blinova, Marge Muna, Margit Heinlaan, Aljona Lukjanova, Anne Kahru

**Affiliations:** 1 Laboratory of Environmental Toxicology, National Institute of Chemical Physics and Biophysics, Tallinn 12618, Estonia; irina.blinova@kbfi.ee (I.B.); margemuna@gmail.com (M.M.); margit.heinlaan@kbfi.ee (M.H.); aljona.lukjanova@kbfi.ee (A.L.); 2 Estonian Academy of Sciences, Tallinn 10130, Kohtu 6, Estonia

**Keywords:** ecotoxicology, bioaccumulation, nanomaterials, rare-earth elements, bibliometric analysis, safety

## Abstract

Lanthanides (Ln), applied mostly in the form of nanoparticles (NPs), are critical to emerging high-tech and green energy industries due to their distinct physicochemical properties. The resulting anthropogenic input of Ln and Ln-based NPs into aquatic environment might create a problem of emerging contaminants. Thus, information on the biological effects of Ln and Ln-based NPs is urgently needed for relevant environmental risk assessment. In this mini-review, we made a bibliometric survey on existing scientific literature with the main aim of identifying the most important data gaps on Ln and Ln-based nanoparticles’ toxicity to aquatic biota. We report that the most studied Ln for ecotoxicity are Ce and Ln, whereas practically no information was found for Nd, Tb, Tm, and Yb. We also discuss the challenges of the research on Ln ecotoxicity, such as relevance of nominal versus bioavailable concentrations of Ln, and point out future research needs (long-term toxicity to aquatic biota and toxic effects of Ln to bottom-dwelling species).

## 1. Introduction

In the periodic table, the lanthanides (Ln) comprise 15 metals with atomic numbers from 57 (La) to 71 (Lu). Together with Sc and Y, they form the group of rare earth elements (REE), as defined by the International Union of Pure and Applied Chemistry (IUPAC). Ln (mostly in the form of oxides) is widely used in modern technologies, e.g., catalysis, electronics, cell phones, LED light bulbs, wind turbines, electric cars, fuel cells, and fuel additives, due to their unique magnetic, phosphorescent, and catalytic properties [1,2]. Electric cars, for instance, contain remarkable quantities of lithium and neodymium, wind turbines neodymium and dysprosium, and solar cells contain several Ln [3]. Due to that, REE have been named “the vitamins of modern industry” [4].

Ln are often applied as manufactured nanoparticles (NPs, i.e., particles with at least one dimension less than 100 nm) [5], usually in the form of Ln oxides, and used e.g., in solid oxide fuel cells and in gas separation membranes. One of the most widely used Ln oxide NPs is CeO_2_ with the global estimated market volume at 2016 of 9100 t and the main application areas energy storage and polishing [6]. Gd_2_O_3_ NPs may replace the Gd-chelates currently used as contrast agents in magnetic resonance imaging due to their higher performance [7]. In addition, La_2_O_3_ NPs [8] CeO_2_ NPs [9,10,11], Gd_2_O_3_ NPs [12], and Tb-based [13] and Ln-doped NPs [14] also have potential as antimicrobials.

A detailed review on technologies and environmental impact concerning Ln has recently been published by [2] and it is recommended for further and more detailed information. Apart from technological applications, Ln have been used as micro-fertilizers [15] and may also end up in the environment as side-products of application of phosphorous fertilizers [16]. Ln-based NPs can reach aquatic ecosystems via several pathways, e.g., by exhaust emissions, leaching from coatings and paints, and via effluents from waste water treatment plants from industry [17]. Approximately 3% of the produced CeO_2_ NPs have been estimated to reach the aquatic environment [17], where they mainly accumulate in sediments [18]. However, the presence of these NPs at concentrations up to 5.2 ng/L has already been recorded in Dutch surface waters [19]. Ln-containing electronic waste problems are also increasing and need addressing [2].

Gadolinium is perhaps one of the most widely studied lanthanide in the environment, due to the wide use of Gd chelates as contrast agents for MRI [20]. A substantial increase in Gd levels in water bodies over the past decades has been reported [21,22]. The increasing application and registration of ‘anthropogenic’ anomalies of Ln in different environmental compartments indicate that Ln are the new emerging contaminants [23]. Thus, relevant data on environmental fate and (eco)toxicity of Ln are needed for the evaluation of the potential hazards of Ln contamination of the environment and, in particular, of the aquatic ecosystems. It could be assumed that, analogously to metal-based nanomaterials (CuO, ZnO, nAg) [24,25,26], soluble Ln compounds leached from Ln-based NPs induce the observed toxic effects. Indeed, in the case of Dy_2_O_3_ NPs, dysprosium ion was the main contributor to the overall toxicity of the Dy_2_O_3_ NPs towards *E. coli* [27] and dissolved Ce-ions ‘explained’ the toxicity of CeO_2_ NPs to algae *Chlamydomonas reinhardtii* [28]. Additionally, it was shown that Ce-ions (but not CeO_2_ NPs) were taken up by *C. reinhardtii* [29]. Thus, the knowledge on (eco)toxicity of Ln ions is an integral part of safety assessment of Ln-based nanomaterials. However, the level of knowledge on environmental concentrations, hazardous levels, speciation, and bioaccumulation properties of Ln ions is remarkably lower [30,31] when comparing with widespread toxic contaminants, such as Pb, Cd, Hg, As, Cu, and Zn [32]. During the last decades, both beneficial and adverse biological effects of Ln on different groups of organisms have been reported [30,33,34,35]. Most studies have focused on their potential effects on humans, soil organisms, and plants, as Ln are widely used in medicine and agriculture (fertilizers). Specifically, it has been reported that increasing Ln concentrations in the environment may cause their accumulation in humans necessitating the need for long-term studies and observations [36,37,38,39]. Although recently comprehensive reviews on e.g., ecotoxicity of certain Ln have been published, for example, on lanthanum by Herrmann et al. [40] and on CeO_2_ NP by Dahle and Arai [41], and it has been shown that both Ln salts and Ln-based NPs can negatively affect aquatic biota [30]; overall data on aquatic organisms are scarce [42,43]. A few attempts have been made to determine the safe levels for Ln in surface waters [40,44], but it remains a challenge due to the lack of reliable data.

We intended to provide an overview on accumulated scientific information on lanthanides/Ln compounds’ environmental safety while considering the recent industrial developments and the safety information for chemicals and materials introduced to the market. This overview is mainly based on bibliometric survey on existing scientific literature with the main aim to identify the most important data gaps on Ln and Ln-based nanoparticles’ toxicity to aquatic biota that need to be addressed in the future studies. However, collection of the toxicity values available for Ln was not the target of this mini-review. We also discuss the main challenges of ecotoxicologica evaluation of Ln (interpretation of the toxicity results given the bioavailable fraction often remains unknown) as well as the applicability of laboratory test results for evaluating the risks of environmental Ln contamination. We chose Web of Science database (Clarivate Analytics) as a main source of information for our survey, while assuming that this database reflects both the scientific developments in technologies, as well as in hazard evaluation.

## 2. Existing Ecotoxicological Knowledge on Lanthanides: Identifying the Data Gaps

### 2.1. Choice of the Key-Words for the Search in WoS (Web of Science)

According European Union (EU) Regulation REACH (Registration, Evaluation, Authorisation, and Restriction of Chemicals) the safety assessment must be performed for new and existing chemical compounds on the EU market [45]. As a rule, the standardized toxicity tests (by OECD, ISO, US EPA, ASTM) should be used for ecotoxicity evaluation of chemicals [46], whereas the number and type of the bioassays depend on the chemical’s annul production/sales volume. In general the annual production exceeding 1 t needs data on short-term toxicity to invertebrates (such as *D. magna* acute test) and/or growth inhibition of aquatic plants (e.g., algae or duckweed). When the annual tonnage exceeds 10 t, a fish short-term toxicity test is also needed as well as activated sludge respiration inhibition test. From annual production exceeding 100 t, long-term toxicity tests on invertebrates, fish and bioaccumulation assays are mandatory and from annual tonnage of 1000 also long-term toxicity assays with benthic organisms must be performed. The above-described mandatory tests are a proxy of the aquatic ecosystem that is composed of organisms from different food-chain levels (producers, consumers, and decomposers).

REACH has also issued additional toxicity testing guidelines for low solubility substances (e.g., poorly soluble Ln-based NPs [18]), for which (i) long-term tests on zooplankton or fish, (ii) toxicity to benthic organisms, and iii) an additional sub-lethal endpoint should be used [46]. Thus, information on the Ln toxicity was collected on the basis of the above described recommendations while using the key-words, including organism groups (aquatic macrophytes and microalgae, invertebrates, fish, benthic organisms) of REACH priority. In addition to acute toxicity data, long-term toxicity and bioaccumulation data were studied. Moreover, microorganisms were added to cover another important ecological organism groups in the aquatic environment. The data contributed by the authors of this paper were not omitted if not indicated differently.

### 2.2. Information on Different Ln Compounds: WoS

**Figure 1:**
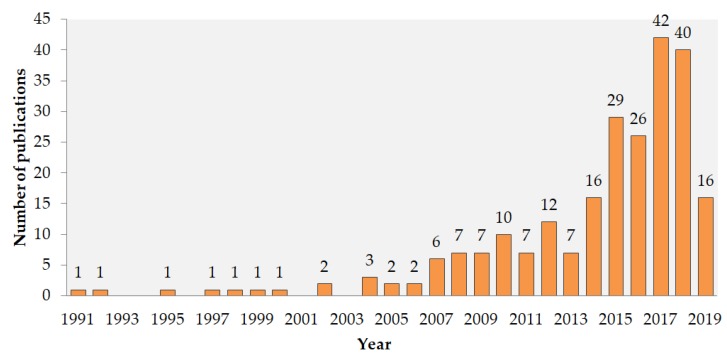
Evolution of information on environmental hazard of Ln according to the number of papers in WoS (for the keywords and raw data, see Table 1). The search was conducted on 4 June 2019.

**Figure 2:**
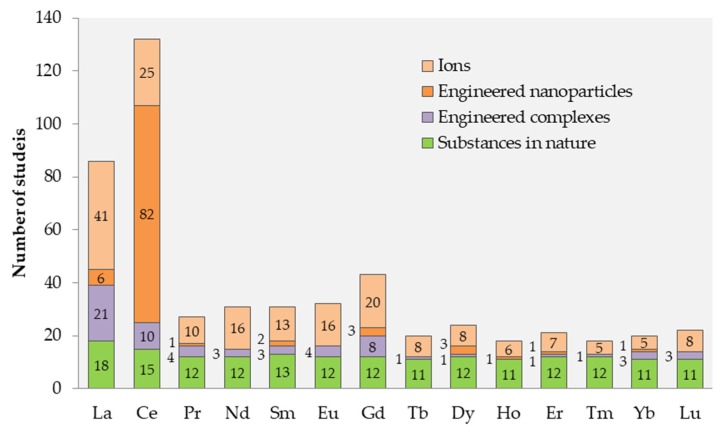
The number of papers for individual Ln compounds in the chemical form of: ions, nanoparticles, complexes or as present in the natural environment (monitoring data). The number of papers is indicated inside or left to the respective column. The pool of initial data analysed in this Figure is described in Table 1 (reviews have been omitted).

**Figure 3:**
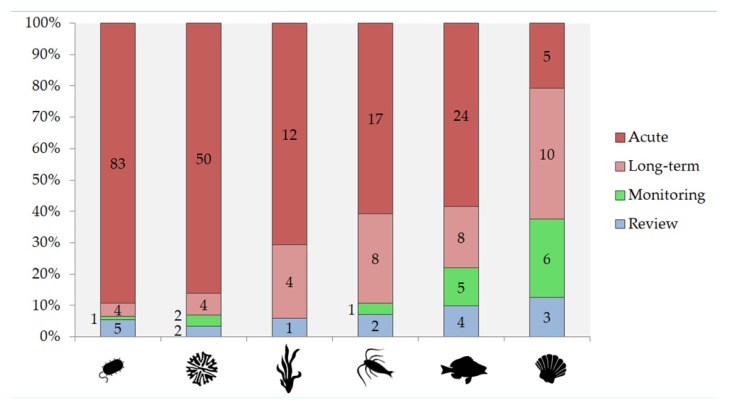
Share of publications on ecotoxicity of Ln based on exposure time: (i) only acute (<14 days) toxicity data, (ii) studies including long-term (≥14 days) experiments, (iii) monitoring studies where data were collected from natural populations, and (iv) reviews. The publications are given separately for different organism groups (see Table 1) from left to right: microorganims, phytoplankton, macrophytes, zooplankton, nekton, and benthos. The number of papers within each category is given inside or left to the respective column.

**Figure 4:**
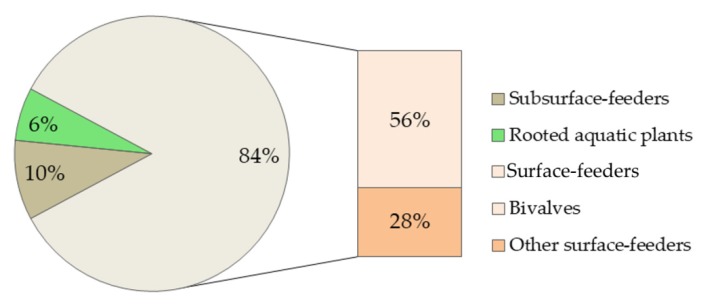
The number of studies including different benthic organism groups based on the data from bibliometric search (Table 1) for macrophytes and benthos (reviews omitted). Altogether 32 species from 23 papers (21 papers on benthos and two papers on macrophytes). Subsurface-feeders included oligochaetes and amphipods *Corophium volutator*. “Other surface-feeders” were collector-feeders (insect larvae, crustaceans, snails) and predators (insect larvae, mites).

**Figure 5:**
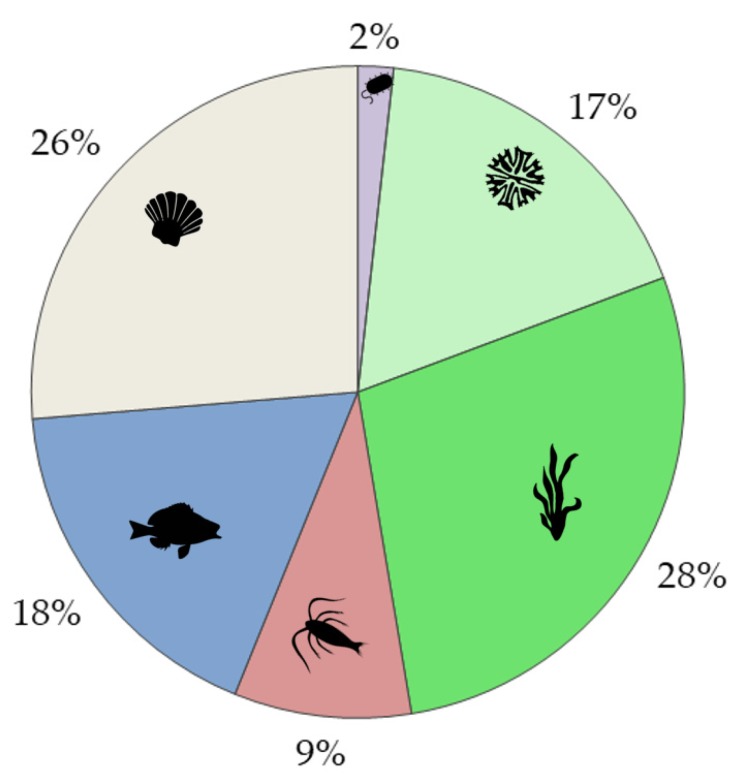
The percentage of organism groups (microorganisms, phytoplankton, macrophytes, zooplankton, fish, and benthos) used in bioaccumulation studies (reviews omitted) based on the bibliometric information on bioaccumulation (see Table 1). 57 organisms were used in the bioaccumulation studies of 45 papers.

The search for information on ecotoxicity of different lanthanides (general search for REE and Ln and for individual Ln from La-Lu) was performed in WoS on 4 June 2019, and the results of the search and the respective search terms (key-words) are presented in Table 1. Altogether, the following organism groups were addressed: microorganisms, phytoplankton, macrophytes, zooplankton, nekton, and benthos. In addition, the number of papers covering both toxicity and bioaccumulation of Ln was separately tabulated (Table 1). This pool of papers (altogether 241 papers, many of which reoccurring in different searches) was used for the analysis of the evolution of the research since 1991 (Figure 1), distribution of the research between different forms of Ln (ions, nanoparticles, complexes, substances in nature) (Figure 2); acute or chronic toxicity data (Figure 3). Information on environmental hazard for benthic organisms (Figure 4) and bioaccumulation (Figure 5) was separately analysed.

As seen in Figure 1, the majority (~90%) of the information on environmental hazard of Ln has been published within the past 10 years. La and Ce were the most studied elements within Ln (Table 1, Figure 2), as also shown by others [43,47]. This can be, in particular, explained by the application of La-based compounds in waterbodies to reduce bioavailable phosphorus to manage noxious cyanobacterial blooms [48]. Element-wise, Pagano et al. (2015) showed the same trend concerning information on human and animal health: Ce, La, and Gd were most studied Ln [36]. Studies on environmental hazard of Ce mostly concerned Ce-based NPs (Figure 2), assumingly due to their wide use as the car-exhaust catalysts and fuel additives [17,41]. The effect of ions was studied in up to half of the studies for all elements except Nd that has been studied more in the form of Nd-ions (Figure 2). For the Ln of higher atomic mass (Tb-Lu), monitoring the data of Ln concentrations in natural environment was dominating (Figure 2).

### 2.3. Analysis of the Existing Information Describing the Environmental Hazard of Ln

#### 2.3.1. Acute and Long-Term Exposure

Long-term toxicity data were best represented among the studies with benthic organisms (40% of the studies) (Figure 3), which was probably due to the abundance of standardised long-term test protocols for these organisms. Long-term studies on Ln were relatively well represented in the publications on zooplankton (29%) and fish (21%), both of which have OECD long-term protocols available and are especially high priority in the REACH legislation. OECD *Daphnia* sp. reproduction test was the most commonly used test for evaluating long term effects of Ln for zooplankton. Macrophytes also had a relatively high proportion (24%) of long-term toxicity data, possibly due to the fact that macrophytes have mainly been used for bioaccumulation (usually long-term) experiments (Figure 3). Long-term toxicity studies on Ln using unicellular organisms were rare (4% of the studies with microalgae and 4% with other unicellular organisms/microorganisms). Most of the microorganisms’ studies concerned the antimicrobial efficiency of engineered (nano)materials.

#### 2.3.2. Information on Toxicity of Ln for Benthic Organisms

Although Ln and Ln-based NPs both tend to accumulate in the sediments [18], only 10% of all the studies (altogether 24 papers on Ln) covered benthic organisms (Table 1). There was just one laboratory study on La [49] and one monitoring study on La from modified bentonite [50], which included macrophytes (*Elodea nuttallii*) with roots growing in the sediment. Most of the experiments and monitoring studies on Ln were conducted with bivalves and other sediment surface feeders (Figure 4). Although filter-feeding bivalves are exposed to contaminants inside the sediments [51], subsurface-feeders, mainly feeding by digesting sediments, are still considered to be better indicators of toxic effects of settled forms of Ln [46,52]. However, only few studies included sediment-ingesting benthic organisms (oligochaets, sediment-dwelling amphipod *Corophium volutator*).

Element-wise, the studies mostly concerned La, Ce, Eu, Gd, and Sm, with no individual Ln ecotoxicological studies on nine Ln (Pr, Nd, Tb–Lu) (Table 1).

#### 2.3.3. Bioaccumulation Studies

21% of the aquatic ecotoxicity publications on Ln (altogether 51 papers) included information on the bioaccumulation of Ln (Table 1). Most of these studies used macrophytes and benthos (mostly bivalves) as test species (Figure 5). No bioaccumulation data were found for five Ln (Nd, Tb, Dy, Ho, Lu; Table 1).

Altogether, there were seven studies that followed all of the major REACH recommendations, e.g., included long-term bioaccumulation studies with benthic organisms. These studies exposed bivalves [33,53,54,55,56], crayfish [57], chironomids [50], and rooted aquatic plants [50] to Ln ions or their complexes. Only one study concerned Ln-based NPs (CeO_2_) [58] examining accumulation in a food web that included bivalves, snails, and benthic shrimps. As a rule, the groups of organisms that were mostly used in the bioaccumulation studies were macrophytes (28%) and bivalves (26% of papers; Figure 5). In addition to exposure studies, eleven bioaccumulation studies were based on the monitoring of natural benthic populations [53,59,60,61,62,63,64,65,66,67,68] that could also be considered to be long term bioaccumulation studies. Similarly to laboratory exposure studies, bivalves were the predominant test species in the monitoring studies [53,59,61,62,63,64,66,67,68]. In addition to bivalves, snails [59], crustaceans [59,60,61], sea urchins [63], insect larvae [59,63], mites and oligochaets [59], and rooted plants [65] were analysed.

## 3. Environmental Safety Assessment of Ln Compounds

The information on (i) potential hazard to living organisms (toxicity values that were obtained from laboratory testing and bioaccumulation potential) and (ii) environmental exposure levels (predicted environmental concentrations) are the key data for environmental safety assessment of chemicals [46]. For Ln, there are knowledge gaps in both of these data groups. Below, we will mostly focus on the first aspect—hazard data—briefly summarizing existing information on the hazard of Ln to aquatic species and highlight the main knowledge gaps. More detailed overviews of Ln toxicity values are available in other reviews [30,40,43,47,69].

### 3.1. Potential Hazard of Ln to Aquatic Ecosystems: State of the Art

#### 3.1.1. Toxicity to Aquatic Biota

The available information shows that Ln based (nano)particles (mostly CeO_2_) may pose a threat to aquatic biota, but the majority of the experiments reported effect concentrations exceeding 10 mg Ce/L. For example, ciliates were tolerant to CeO_2_ NPs at concentrations up to 200 mg/L, depending on the composition of the multispecies communities in a 64-day experiment [70]. Analogously, no toxicity of CeO_2_ was observed for protozoan *Tetrahymena thermophila* (24 h EC_50_ > 100 mg/L) and bacteria *Vibrio fischeri* (30-min. EC_50_ > 500 mg/L) although the particles were producing reactive oxygen species (ROS) in abiotic conditions [71]. No adverse effects of CeO_2_ NPs on chironomid development, growth, or emergence were reported upon exposure up to 100 mg/L, despite the fact that ingestion of the particles by organisms was recorded [72]. 10-day CeO_2_ NPs exposure to up to 100 mg/L did not induce mortality for sediment-dwelling amphipod *Corophium volutator*, while DNA damage and oxidative stress were induced at exposure concentration of 12.5 mg/L [73]. CeO_2_ NPs induced significant adverse biological effects in long-term studies with crustaceans and fish at 10–100 mg Ce/L [74,75]. It also has been shown that Ln can change the microbial community’s structure: a 15-day exposure to CeO_2_ NPs increased the proportion of algae and decreased the proportion of bacteria in the biofilm [76]. The toxicity study of nine (doped) lanthanide oxides for crustaceans *Daphnia magna* (48 h immobilisation test) and algae *Raphidocelis subcapitata* (72 h growth inhibition assay) showed that the toxicity of the most toxic compounds was due to toxic heavy metals that were used as dopants that were shed into the test environment (e.g., Ni from LaNiO_4_) [77,78]. Thus, Ln based NPs are neither the most toxic NPs [79,80] nor safe for the aquatic biota.

As a rule, Ln based (nano)particles have proven to be remarkably less toxic than the corresponding soluble Ln salts [71,77]. However, it is very difficult to differentiate between the toxic effects of ions and particles due to the limited knowledge on toxicity of Ln ions and Ln behaviour in the test medium. For example, Sm and Ho oxide NPs were toxic for *Hydra attenuata* (96 h EC_50_ 0.1–1 mg/L), but in crustaceans *Thamnocephalus platyurus* assay, the same NPs induced no toxicity (24 h LC_50_ > 100 mg/L) [81]. However, for Ln-based NPs with low dissolution rates, e.g., CeO_2_, particle-induced toxicity might be more relevant [75,82]. The dissolution of Ln-based NPs might occur at nano-bio interface and be an important cause of toxic effects [11,83,84,85]. Other particle-specific effects include physical effects after ingestion by, or adsorption on, the organism [86,87,88], induction of oxidative stress [89,90], and membrane damage [71,91,92,93,94], along with entrapment of unicellular organisms into NPs agglomerates [28,77].

Most data on the Ln salts’ toxicity to aquatic organisms results from the acute tests (Figure 2), whereas the results remarkably vary. For example, acute (48 h) Ln toxicity (EC_50_) to crustaceans *D. magna* that was calculated based on the measured total concentration in test medium ranged from 0.2 to 24 mg Ln/L [40,44,95] and EC_50_ calculated on the measured dissolved Ln concentrations ranged from 0.04 to 1.2 mg Ln/L [96,97]. Movement inhibition-based EC_50_ for oligochaets exposed to Ln salts was 9.6–12 g Ln/L, which is a very high concentration but still similar to the results of toxic metals, such as Cd and Ni in the same test conditions [98]. Long-term studies with benthic filter feeders, such as pearl oysters, have shown that food-borne exposure to Eu^3+^ and Eu complexes modified the microstructure and colour of pearls produced by pearl oysters indicating the possibility of Eu being metabolised similarly to Ca [99]. Additionally, it was shown that Ln-rich river sediment induced mortality of benthic ostracods [100]. Long-term exposure of *Daphnia* to natural REE-enriched mine tailing leachates resulted in a larger number of offspring that was smaller in size when compared to the control, indicating that Ln might induce adverse effects in natural conditions [101]. The gills and liver of fish may also be adversely affected upon long-term exposure to CeO_2_ NPs [102].

Studies with macrophytes also yielded highly variable results. Chlorophyll reduction and oxidative damage has already been observed at 1.4–2.8 mg Pr/L in duckweed *Spirodela polyrrhiza* with Ln mainly deposited in the cell wall [103,104]. On the other hand, the growth of another duckweed species—*Lemna minor*—was promoted by the presence of Ce salts at concentrations up to 139 mg Ce/L and only decreased at higher concentrations of Ce [105]. The long-term (17–21 days) studies with cyanobacteria showed hormesis at concentrations up to 0.1 or up to 0.5 mg Ln/L after exposure to LaCl_3_ [106] and CeCl_3_, respectively [107]. Higher concentrations induced a decline in growth, reproduction, chlorophyll a content, and K and Mg content. Even though Ln are also used as Ca channel blockers to study uptake routes of other heavy metals, Ca concentrations in cyanobacteria increased with increasing LaCl_3_ exposure levels [106].

Factors that may be the cause for high intraspecific variation of Ln toxicity values will be discussed below (Section 4).

#### 3.1.2. Bioaccumulation of Ln

It is difficult to draw conclusions regarding the Ln bioaccumulation results, since the test design (test organisms, exposure conditions, test duration, type of Ln compounds) in different studies varied considerably (Section 2.3.3). For example, it was shown that Gd accumulated approximately 100-fold more when applied as GdCl_3_ when compared to application as Gd-based contrast agent common in hospital waste waters [33]. Studies with bivalves showed that the La and Ce contents were higher in mussels (mean 0.041–0.069 mg/kg) than in oysters (0.012–0.021 mg/kg) [67]. Anthropogenic and geogenic Ln may accumulate differently. Anthropogenic Gd from contaminated sites was not incorporated into river bivalve shells, whereas geogenic Gd was [64], thus confirming the experimental results of [34]. However, anthropogenic La and Sm were bioavailable and accumulated in the shells similarly to geogenic ones [65].

According to most publications, Ln may be classified as elements with low bioaccumulation potential in aquatic organisms. The highest reported bioconcentration and bioaccumulation factor values for bivalves were between 23 and 357 [34,54,55,56], which are well below 2000—the threshold to classify substances as bioaccumulative by REACH legislation (Annex VIII) [45]. Freshwater crayfish accumulated La from La modified bentonite, mainly in gills resulting in 122-fold increase when compared to the control (182 µg/g) and in the carapace (18 µg/g), indicating La uptake by gills [57]. Similarly, La content in rooted plant *Elodea nuttallii* increased up to 127-fold when compared to the control plants during the first growing season after application of La-modified bentonite. The concentrations remained up to 112-fold elevated as compared to the control for at least two years after Ln-modified clay application proving the high accumulation potential for plants. The concentrations in *E. nuttallii* reached up to 871 mg/g La in its tissues within a month from application of La modified bentonite into the test environment, but accumulation in filter-feeding chironomid larvae was insignificant [50].

A relatively high Ln content in biota has also been reported. Ln (together with Sc and Y) concentration in soft tissue of bivalves reached 1.6 mg/kg in studies by Rodriguez-Hernandez et al. [66]. Marine crabs in fertiliser-polluted waters accumulated waterborne Ln in the shell (2.5 mg/kg) and foodborne Ln in the claw muscles (0.44 mg/kg) [60]. Study on Ln pollution-exposed crabs showed that Tm and La were also found in the exoskeleton [61]. Ln accumulation (0.02–12.2 mg/kg) in Portugese freshwater mosses led to a bioconcentration factor of up to 1.1 × 10^6^ being the highest for La and Ce, whereas all 13 studied REEs could be classified as bioaccumulative [65].

The biomagnification potential of Ln could still be considered to be limited, according to available literature. Indeed, even biodilution of Ln has been observed in natural ecosystems [59,63]. For example, the Ce concentrations were lower in higher food chain level (fish) than in lower food chain level organisms in bioaccumulation experiments with CeO_2_ NPs in the constructing freshwater ecosystem [58].

### 3.2. Environmental Exposure Levels of Ln

Assessment of the Ln release into the environment and the resulting accumulation in the aquatic ecosystems should consider different emission sources and pathways, as well as the fate of anthropogenic Ln in various environmental matrices. This is a very difficult task, given the variety of Ln industrial applications and lack of the experimental data on Ln behaviour in aquatic ecosystems [2,41]. Notably, there are knowledge gaps, even on Ln release from the mine wastes [108] —the oldest Ln pollution source. Therefore, only the direct measurement of Ln in different environmental compartments allows for evaluating the contamination trends due to the lack of a working life-cycle material flow and environmental fate models for Ln. The data obtained show that the surface waters are generally characterized by very low (<ng/L to 200 ng/L) Ln concentrations [109,110,111,112,113,114,115,116,117]. However, the higher levels of Ln have also been reported: sum Ln concentrations in the Syr Darya River ranged from 15.1–28.3 µg/L [118] and concentrations of Ln in stream waters (Eastern Canada) from <5 to 11,540 ng/L, with an average of 253 ng/L (n = 498) [119]. At very polluted sites, Ln concentrations may increase up to 78 μg/L [114,120] and, in exceptional cases, e.g., in acid mine drainage waters or after lake restoration while using Ln-modified bentonite clay, Ln concentrations may even reach 15 mg/L [116,121,122,123,124,125]. Thus, comparison of the Ln concentration in the surface waters and reported toxicity values allows for concluding that, although even in contaminated waters Ln concentrations are still lower than the reported toxic concentrations for aquatic organisms, in certain cases (e.g., in the treated water bodies or mine waste water) Ln may already disturb normal function of ecosystem.

## 4. Uncertainties in Evaluation of Potential Hazard of Ln Compounds to the Aquatic Organisms

The main problems that complicate the use of the laboratory ecotoxicity test results for the realistic chemicals’ safety assessment are similar to Ln compounds and other types of metal-based nanoparticles, namely (i) the interpretation of the obtained toxicity values and (ii) ecological relevance of the data that were obtained in the laboratory tests.

### 4.1. Behaviour of Ln in the Test Environment

Correct reporting of real exposure concentrations is the most important problem in the interpretation of the toxicity test results. The reported effect concentrations for Ln compounds may be calculated either on the basis of nominal or measured concentrations in the test media (total or dissolved), making the comparison of the results from different studies complicated or impossible (see Section 3.1.1). The same holds true for the extrapolation of the laboratory data to aquatic ecosystems (from lab to field).

It is known that speciation and, as a result, the bioavailability of Ln compounds (added in soluble or poorly soluble form) in the test environment, mostly depends on the chemical composition of the test medium. Indeed, variation of the Ln toxicity values, depending on the test medium, has been demonstrated in several studies [95,96]. For metal-based nanomaterials, the main processes affecting bioavailability in the test medium are aggregation, sedimentation, and dissolution. Ln-based NPs can be stabilised by phosphates [28] and by organic matter [126]. The stabilising effect has shown to be weaker for CeO_2_ NPs already stabilised with polymers by the manufacturer [127]. Suspended CeO_2_ NPs were more toxic to zooplankton than settled particles [128]. The stabilising effect of NPs by organic matter also increased the uptake of citrate-coated CeO_2_ in fish [129], but decreased toxicity of uncoated CeO_2_ to fish [130]. The presence of Fe, on the other hand, increased microorganism-CeO_2_ interactions and, thus, the toxicity [131].

Ln-based NPs [131] and, especially, their agglomerates caused flocculation of algae [28,77] similarly to Ln salts. Ln ions, in turn, tend to form insoluble or poorly soluble complexes in most ecotoxicity test media [132,133]. The presence of phosphates leads to Ln precipitation [28,71,134,135]; chlorides may increase the solubility of Ln compounds [121]; high water hardness causes the precipitation of Ln, destabilisation and coagulation of Ln colloids; Fe and Al ions promote the formation of soluble Ln species [111,125,136]. The presence and chemical composition of dissolved organic matter may also significantly affect Ln bioavailability [137,138,139]. Ln internalisation is lower in the presence of organic matter [138], but complexes with small organic molecules can be taken up by the cells of aquatic plants [105], fish [140], and algae [88,141]. The test medium has strong impact on Ln toxicity, as insoluble Ln salts may cause reducing Ln bioavailability, and thus toxicity, as mentioned above. For example, reduction of the phosphate concentration (nutrient sequestration) in the test medium by Ln was the main mechanism for algal growth inhibition reported by Joonas et al. [77]. In addition to the composition of the test medium, the speciation of Ln remarkably depends on the used nominal concentration and exposure duration [95,139,142]. Moreover, Ln ‘ionic’ complexes are labile and, thus, Ln bioavailability might significantly vary during experiments [143].

### 4.2. Ecological Realism of the Laboratory Toxicity Test Results

Low ecological relevance of standardized ecotoxicity testing methods is increasingly discussed [144,145]. The main weaknesses that hamper extrapolation of laboratory test results to aquatic ecosystems are common for all chemicals/pollutants [146]. However, in the case of Ln, they are especially important, mostly due to the speciation issues that are described above. In addition, in laboratory tests, Ln/Ln NPs behaviour significantly differs from the natural conditions, due to (i) too high (environmentally irrelevant) exposure concentrations and (ii) environmentally irrelevant chemical composition of the test media (e.g., artificial fresh water lacking organics). Thus, a shortage of information on the Ln behaviour in the different exposure medium and natural waters make the correct interpretation of the toxicity test results very problematic.

It has been previously shown that, for the hazard evaluation of Ln, acute toxicity data are not reliable due to very high nominal concentrations and the very short exposure duration used in acute toxicity assays [95], but most of the publications on Ln toxicity present results from acute tests (Figure 2). Long-term experiments are also much more informative as the transformation of Ln NPs in the environment might change their bioavailable fraction [147].

Another limitation of the laboratory testing is the small number of tests species usually used in chemical safety evaluation. Moreover, in the case of Ln compounds, benthic organisms should be more represented. For example, fish take up CeO_2_ NPs, largely by the gastrointestinal tract [148], thus making benthic and bottom-feeding fish more prone to the potential harmful effects of CeO_2_ via NPs uptake [149]. However, information on potential Ln toxicity to benthic organisms is very limited (Figure 2 and Figure 4).

### 4.3. Ln as a Uniform Group of Elements

It could be assumed that the toxicity of individual Ln to biota is similar due to similar chemical properties [150], and from the environmental safety point of view, Ln may be considered as a uniform group of elements. Indeed, most of the studies support this hypothesis. Tai et al. [151] evaluated the toxicity of 13 Ln to unicellular algae *Skeletonema costatum* and showed that the growth inhibitory effects of all these elements were similar, i.e., not dependent on Ln: the 96 h EC_50_ values were approximately 29 micromol/L. Five Ln nitrates (Ce, Gd, La, Nd, and Pr) showed very similar toxicity to unicellular alga *Rapidocelis subcapitata* [77], protozoa *Tetrahymena thermophila* [71], and crustacean *Daphnia magna* [95]. However, for *V. fischeri,* the toxicity values of these five Ln varied remarkably, ranging from 3 mg/L for Gd to 21 mg/L for La [71].

In general, the accumulation of Ln is in accordance with their concentrations in nature [59], although Ln of lower atomic mass tends to accumulate in slightly higher concentrations. Indeed, Ln of lower atomic mass were consistently more concentrated in natural benthos samples of bivalves, in sea urchins, freshwater benthos [63], crabs [60], soft tissues of mussels [62], as well as in freshwater mosses [65]. Additionally, the Ln-tolerant strains of bacteria preferred to take up Ln of lower atomic mass from Ln-containing acid mine drainage [152]. In another acid mine drainage exposure experiment, however, Ln of medium atomic weight accumulated in bivalves more than light or heavy ones [53]. Variation in toxicity or the accumulation of individual Ln tested at the same conditions may be explained by slightly different chemical behaviour [41] and, consequently, their bioavailability to the test organisms.

## 5. Summary

Analysis of published information regarding the potential hazard of Ln compounds to aquatic ecosystems showed that the current accumulated knowledge on Ln toxicity and behaviour in the complex systems is too scarce to support the reliable environmental safety assessment. The main data gaps and recommendations for further investigations are as follows:

- The most ecotoxicologically studied Ln are Ce and Ln. Practically no information was found for Nd, Tb, Tm, and Yb. More attention in scientific research could be drawn to Ln with lower atomic mass, as they are more abundant and tend to bioaccumulate more than heavy Ln.
- There is a considerable lack of long-term ecotoxicity data from environmentally relevant exposure conditions (Ln concentrations and test media), although these data are the most relevant for an evaluation of the potential hazard of anthropogenic Ln to aquatic ecosystems.
- As Ln tend accumulate in the sediments, more attention should be paid to the adverse effects of Ln to bottom-dwelling species, especially to sediment-digesting ones that are underrepresented in the current literature.
- Although the current environmental concentrations of Ln are still too low to cause adverse effects, remarkable bioaccumulation of Ln in some aquatic plant species is a warning sign. Therefore, additional information on the Ln bioaccumulation potential at all food chain levels is needed.
- There is an urgent need for additional knowledge on the behaviour of Ln in the aquatic environment.

## Figures and Tables

**Table 1:** Information on environmental hazard of individual lanthanides for main aquatic organism groups: number of papers in Web of Science (WoS), including 16 reviews. Search in WoS was made on 4 June 2019 and it covered Title, Abstract, Author Keywords, and Editor Keywords. The search terms are presented and explained in the footnotes.

Ecological Group	Ln, REE ^8^	La	Ce	Pr	Nd	Sm	Eu	Gd	Tb	Dy	Ho	Er	Tm	Yb	Lu	Total *
**Microorganims ^1^**	19	11	48	3	1	3	3	5	1	3	1	1	0	1	3	93
**Phytoplankton ^2^**	21	13	28	1	0	4	2	5	1	0	1	1	1	0	1	58
**Macrophytes ^3^**	13	6	5	1	1	0	0	0	0	0	0	1	0	1	0	17
**Zooplankton ^4^**	13	7	14	0	0	1	0	2	0	1	1	1	0	0	1	28
**Nekton ^5^**	17	13	22	1	0	1	2	3	0	0	1	1	0	0	1	41
**Benthos ^6^**	17	8	7	0	0	2	1	3	0	0	0	0	0	0	0	24
**Bioaccumulation ^7^**	37	12	20	1	0	2	3	2	0	0	0	1	1	1	0	51

^1^ (element name) AND (* toxic *) AND (microorganism * OR bacteri * OR protozoa OR protist * OR yeast) ^2^ (element name) AND (* toxic *) AND (phytoplankton OR microalga * OR alga *) ^3^ (element name) AND (* toxic *) AND (macrophyte * OR “aquatic plant *” OR macroalga * OR duckweed OR Myriophyllum) ^4^ (element name) AND (* toxic *) AND (zooplankt * OR microinvertebrate * OR microcrustacea * OR * daphni * OR “D. magna”) ^5^ (element name) AND (* toxic *) AND (fish OR Danio “or “D. rerio”) ^6^ (element name) AND (* toxic *) AND (benth * OR mussel * OR clam * OR oyster * OR oligichaet * OR amphipod * OR chironomid *) ^7^ (element name) AND (* toxic *) AND (* accumulat * OR “body burden” OR * uptake) AND (marine OR aquatic OR aqueous OR * water *) ^8^ (Lanthanide * OR “rare earth”) AND (* toxic *) AND (one of the combinations given above) * Total number of papers found in the literature search. The total number is lower than the sum of the papers found in each individual search as some of the papers occurred repeatedly in the searches.
